# Plant‐expressed Fc‐fusion protein tetravalent dengue vaccine with inherent adjuvant properties

**DOI:** 10.1111/pbi.12869

**Published:** 2018-02-01

**Authors:** Mi Young Kim, Alastair Copland, Kaustuv Nayak, Anmol Chandele, Muhammad S. Ahmed, Qibo Zhang, Gil R. Diogo, Matthew J. Paul, Sven Hofmann, Moon‐Sik Yang, Yong‐Suk Jang, Julian K‐C. Ma, Rajko Reljic

**Affiliations:** ^1^ Institute for Infection and Immunity St George's University of London London UK; ^2^ Department of Molecular Biology and the Institute for Molecular Biology and Genetics Chonbuk National University Jeonju Korea; ^3^ ICGEB‐Emory Vaccine Center International Center for Genetic Engineering and Biotechnology New Delhi India; ^4^ Department of Clinical Infection, Microbiology and Immunology Institute of Infection and Global Health University of Liverpool Liverpool UK

**Keywords:** dengue, vaccine, neutralizing antibodies, human, IgG, Fc‐fusion proteins

## Abstract

Dengue is a major global disease requiring improved treatment and prevention strategies. The recently licensed Sanofi Pasteur Dengvaxia vaccine does not protect children under the age of nine, and additional vaccine strategies are thus needed to halt this expanding global epidemic. Here, we employed a molecular engineering approach and plant expression to produce a humanized and highly immunogenic poly‐immunoglobulin G scaffold (PIGS) fused to the consensus dengue envelope protein III domain (cEDIII). The immunogenicity of this IgG Fc receptor‐targeted vaccine candidate was demonstrated in transgenic mice expressing human FcγRI/CD64, by induction of neutralizing antibodies and evidence of cell‐mediated immunity. Furthermore, these molecules were able to prime immune cells from human adenoid/tonsillar tissue *ex vivo* as evidenced by antigen‐specific CD4^+^ and CD8^+^ T‐cell proliferation, IFN‐γ and antibody production. The purified polymeric fraction of dengue PIGS (D‐PIGS) induced stronger immune activation than the monomeric form, suggesting a more efficient interaction with the low‐affinity Fcγ receptors on antigen‐presenting cells. These results show that the plant‐expressed D‐PIGS have the potential for translation towards a safe and easily scalable single antigen‐based tetravalent dengue vaccine.

## Introduction

Dengue infection poses a significant health risk with almost a hundred million cases worldwide, of which half a million are severe (Bhatt *et al*., 2013), characterized by organ dysfunction, increased vascular permeability, haemorrhagic fever and dengue shock syndrome. To counter this expanding global health threat, better case identification, improved patient management and new preventive strategies are all urgently needed. A major breakthrough in vaccine development occurred recently with the licensing of the CYD tetravalent dengue vaccine in several countries, following two large efficacy trials in Asia and South America (Capeding *et al*., 2014; Watts *et al*., 1998). However, the Sanofi Pasteur produced Dengvaxia (as it is commercially known) is only partially effective against serotype 2 and most importantly contraindicated in children under the age of nine (Hadinegoro *et al*., 2015), due to suboptimal responses in this age population and risk of antibody‐dependent enhancement of infection (ADE). Also, while the vaccine has been cautiously endorsed by the WHO (Wilder‐Smith *et al*., 2016), there remain a number of other issues with this vaccine, including its stability, cost‐effectiveness and affordability (Deen *et al*., 2016; Godoi *et al*., 2017; Harapan *et al*., 2017; Shafie *et al*., 2017).

Meanwhile, several other vaccine candidates have either progressed to clinical trials or are in the late stages of preclinical development. Thus, one of the most advanced alternatives to Dengvaxia is the DENVax vaccine developed by Takeda Vaccines Inc (Brewoo *et al*., 2012; Osorio *et al*., 2011), currently undergoing phase 2 trials in Asia and Latin America (Saez‐Llorens *et al*., 2017). Similarly, the LAV Delta 30 (NIAD/Butantan) dengue vaccine has completed phase 1 clinical trials (Durbin *et al*., 2011) and is about to enter phase II trials. All three aforementioned tetravalent vaccines are based on attenuated viral backbone platforms and while this may be an advantage when comparing their performance in different geographical areas and age populations, there is a certain element of overlap which may lead to common safety, stability and cost‐effectiveness issues. There is a need for additional and preferably complementary vaccine approaches which could be used instead of or alongside one of the viral tetravalent vaccines to achieve a satisfactory level of protection in all subjects.

Several promising nonlive subunit dengue vaccine candidates have been reported, including DNA vaccines (Poggianella *et al*., 2015; Porter and Raviprakash, 2015), nanoparticle formulations (Swaminathan *et al*., 2016) and lipidated dengue antigens (Chiang *et al*., 2016). These and other preclinical candidates currently being considered are necessary for continuous feeding of the dengue vaccine pipeline until an effective vaccine strategy against this global pandemic is finally attained. Our own approach has been to evaluate novel dengue subunit vaccine candidates expressed in genetically modified plants and designed to be inherently self‐adjuvanting (Kim *et al*., 2015, 2016). Many potential biologics have been expressed in genetically modified plants (Arntzen, 2015) and this trend is likely to continue with the increasing demand for cheaper medicines produced at large scales. One of the more successful recent examples reaffirming the growing promise of plant‐made biologics is highlighted by the success story of ZMapp™ as a potentially life‐saving drug during the Ebola outbreak of 2014–2016 (Lyon *et al*., 2014). Importantly, unlike bacteria, plants are capable of performing post‐translational modifications in proteins which makes them amenable for expression of complex proteins such as antibodies and the Fc‐antigen fusion proteins described here.

Fusion proteins based on immunoglobulin Fc domain have received considerable attention over the past two decades, as either therapeutic tools or potential vaccine delivery platform. Currently, Fc‐fusion‐based biologics are some of the biggest profit grossing drugs in pharmaceutical industry (Czajkowsky *et al*., 2012). However, the use of Fc‐fusion proteins in vaccine development has lagged behind. Indeed, several reports describe the use of fusion proteins as vaccine constructs against a number of conditions including infections, allergies and cancer (reviewed in Czajkowsky *et al*., 2012), but to our knowledge, no vaccine based on Fc‐fusion proteins has been licensed yet for any of these conditions.

Nevertheless, Fc‐fusion proteins have considerable vaccine potential, from both an immunogenic and a safety point of view. Not only are they inherently safe as nonlive, protein subunit vaccines, but their potential self‐adjuvanting nature means that the need for exogenous adjuvants is reduced. The targeting of Fc receptors on antigen‐presenting cells (APCs) leads to enhanced antigen uptake and processing, while their polymeric nature provides an additional antigen depot effect, both properties ordinarily conferred by adjuvants in conventional vaccines. Modelled on the early work of Mekhaiel *et al*. (2011), we have previously tested the immunogenic potential of the polymeric immunoglobulin scaffold (PIGS) as a molecular platform for delivery of the dengue virus envelope domain III in mice and found that they were highly immunogenic (Kim *et al*., 2017). Here, we provide important new evidence for the substantial vaccine potential of the fully humanized version of the dengue PIGS (D‐PIGS) that is critical for translation of this technology towards application in humans.

## Results

### D‐PIGS are immunogenic in human adenotonsillar tissue

**Figure 1:**
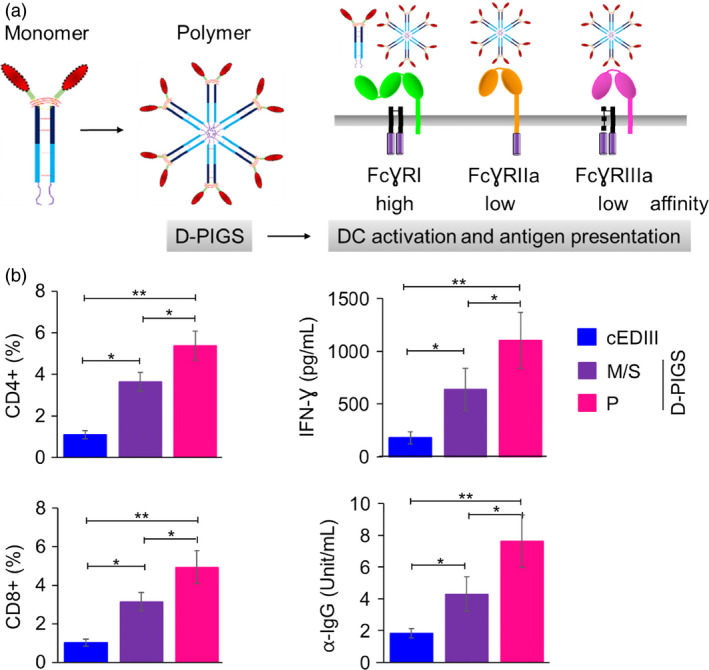
Dengue poly‐immunoglobulin G scaffold (D‐PIGS) induce cellular and humoral immune responses in human adenoid/tonsil cultures. (a) Schematic representation of the monomeric and polymeric human IgG1‐cEDIII fusion protein and its interaction with high and low‐affinity Fc gamma receptors on antigen‐presenting cells; further details of the structure of these molecules are described in Kim *et al*. (2017). Red represents cEDIII domain, while dark and light blue indicate CH2 and CH3 domains of human IgG1, respectively. (b) Immunogenicity of D‐PIGS in human tonsil cultures; shown are flow cytometric data of CFSE staining expressed as percentages of antigen‐specific CD4‐ and CD8‐proliferating cells, IFN‐γ and IgG concentrations in culture supernatants, induced by cEDIII alone, monomer/single chain (M/S) and polymer (P, D‐PIGS). Data are shown as means ± SEM from eight patients. Statistical analysis was performed by ANOVA and Dunnett's test, where *indicates differences <0.05 and **<0.01.

We generated dengue Fc‐fusion proteins based on human IgG1 heavy chain and consensus EDIII domain of dengue envelope protein, by replacing the variable region with the cEDIII and linking it to Cγ2 and Cγ3 domains by a short peptide derived partly from Cγ1 domain and partly from the hinge region. The μtp is genetically linked at the C′‐terminus. This fusion protein can then form monomers or polymers resembling polymeric IgM. One of the key advantages of the Fc‐fusion proteins over free antigens is their capacity to bind to the Fc receptors on APCs and thus enhance antigen uptake and subsequent processing and presentation. Furthermore, while monomeric Fc‐fusion proteins can bind only to the high‐affinity receptor (FcγRI), the polymeric versions (here termed ‘D‐PIGS’) can bind to both high‐ and low‐affinity receptors (Figure 1a), thus substantially enhancing their overall immunogenic potential. We validated this concept by testing the immunogenicity of either antigen alone, the monomeric fusion protein or the D‐PIGS in human tonsillar tissue cell cultures from patients undergoing elective tonsillectomy and with no previous dengue exposure. CD4^+^ and CD8^+^ T‐cell proliferative responses in CFSE‐labelled cell cultures were measured by flow cytometry (gating strategy shown in Figure S6) as well as IFN‐γ production. As shown in Figure 1b, while both the monomer and polymers induces T‐cell responses and IFN‐γ production significantly above that induced by cEDIII antigen alone (*P* < 0.05 and 0.01, respectively), D‐PIGS were significantly more efficient in doing so than the monomers (*P* < 0.05). Moreover, D‐PIGS also induced higher antigen‐specific IgG response in these cultures over 2‐week incubation period. Taken together, these findings show that this fully humanized dengue vaccine candidate is immunogenic in human immune tissues, inducing both cellular and humoral immune responses in the absence of exogenous adjuvants.

### Plant expression and purification of D‐PIGS

**Figure 2:**
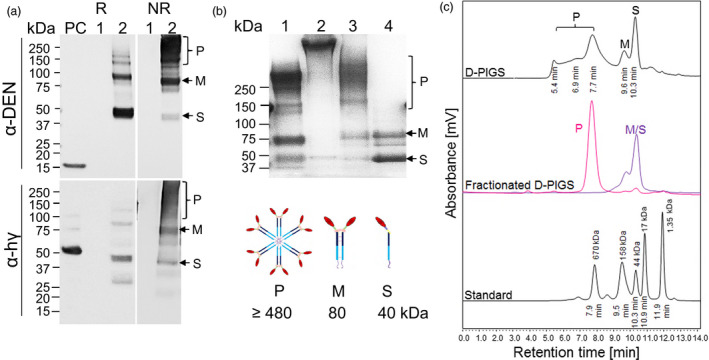
Expression, purification and molecular fractionation of dengue poly‐immunoglobulin G scaffold (D‐PIGS). (a) D‐PIGS were expressed in *Nicotiana benthamiana* plants and the extracts analysed by Western blotting under reducing (R) or nonreducing (NR) conditions using antidengue or anti‐Fc gamma antibodies. ‘PC’ is the positive control (recombinant cEDIII or human IgG1, respectively). Lane 1: wild‐type plant extract; lane 2: unfractionated D‐PIGS. Positions of the single chain (S), monomer (M) and polymers (P) are indicated. (b) SDS‐PAGE and Commassie staining of fractionated D‐PIGS. Lanes: 1. Commercial (Sigma) human sIgA; 2. Commercial (Sigma) human IgM; 3. Polymers and 4. Monomers. The schematics bellow indicate the expected molecular sizes for each fraction. (c) HPLC profile of D‐PIGS. Unfractionated (upper panel) and fractionated (middle panel) D‐PIGS. Indicated retention times were used to estimate the molecular weights of each fraction, based on gel filtration protein standards (bottom panel). The fractionated D‐PIGS were used in immunogenicity studies with tonsillar cultures (Figure 1b).

Molecular cloning of mouse and human versions of D‐PIGS is described previously (Kim *et al*., 2017). Human D‐PIGS were transiently expressed in *Nicotiana benthamiana* plants following infiltration with recombinant agrobacteria and purified by protein A chromatography. This expression system yielded on average 17 mg protein/kg fresh weight plant tissue. Both wild type and a ∆XF glycosylation mutant of *N. benthamiana* plants lacking fucose and xylose glycosylation (Strasser *et al*., 2004) were used, but the latter version was used in all experiments due to its enhanced receptor binding function (Figure S1). D‐PIGS were analysed by SDS‐PAGE electrophoresis and Western blotting, as well as HPLC. Under reducing conditions (R), D‐PIGS yielded a dominant 40‐kDa protein band reactive with both antidengue and anti‐Fcγ antibodies, consistent with a single (S) cEDIII‐Fc‐fusion protein chain (Figure 2a, lane 2). However, under nonreducing conditions (NR), additional protein bands were revealed, including an 80‐kDa band likely representing the monomer (M), and a dominant diffuse band of 160 kDa and above likely representing a mixture of dimers (160 kDa) and polymers (480 kDa expected molecular weight), although this could not be discerned clearly from the gel due to insufficient resolution. Negative (wild‐type plant extract, lane 1) and positive controls (lane ‘PC’) represented by recombinant cEDIII and human IgG1 were included for comparisons.

As the SDS‐PAGE analysis revealed a mixture of monomeric/single chain and polymeric molecular species in the D‐PIGS preparation, we next fractionated the low and high molecular weight forms by HPLC (Figure 2c). The HPLC profile indicated presence of several protein peaks that corresponded to single chain (S), monomer (M) and polymers (P). These could be separated into two main fractions corresponding to a dominant single polymer peak and a mixture of monomer and single chain (Figure 2c, middle panel). The retention times for these protein fractions were compared to those of molecular weight standard proteins (bottom panel) and while M and S eluted as 80‐ and 40‐kDa protein peaks, the polymeric fraction presented as approximately a 680‐kDa protein, which is somewhat higher than the theoretical weight of the D‐PIGS hexamer (480 kDa), even when accounting for 12 carbohydrate chains present on the IgG1‐Fc (equating to approximately 36 kDa). The larger than expected size of the D‐PIGS could be simply an anomaly of the HPLC system and the molecular standards we used or perhaps altered behaviour due to the presence of multiple carbohydrate chains. This is partly supported by SDS‐PAGE analysis of the separated fractions that showed the presence of monomers and single chains in the low molecular weight fraction (lane 4), and a diffuse polymeric protein band (lane 3) which is significantly smaller than the 970‐kDa pentameric IgM (lane 2) (Figure 2b) but bigger than the 380‐kDa human sIgA (lane 1) (Figure 2b). The separated polymeric fraction was stable and showed no degradation or change in the ratio of the two molecular forms (hexamers and higher polymers) upon 24‐h storage at room temperature, +4 or −20 °C (Figure S2).

### Analysis of D‐PIGS binding to Fc gamma receptors

**Figure 3:**
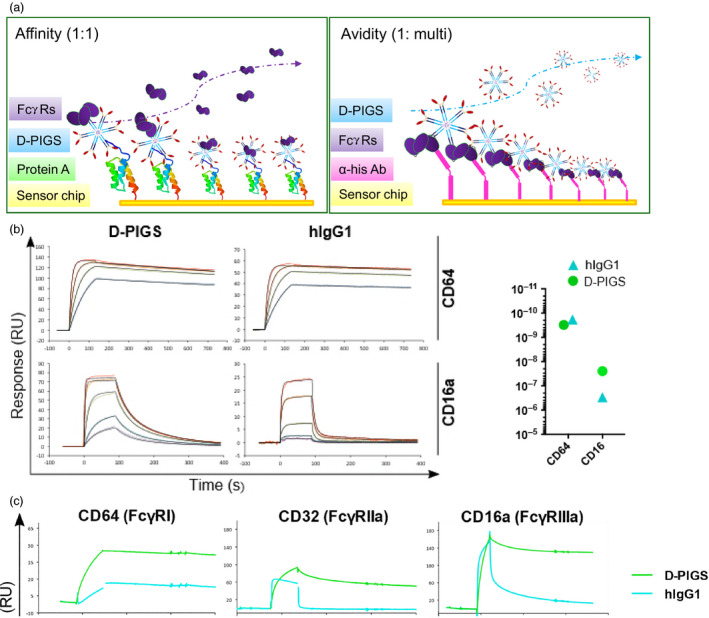
Biacore analysis of binding of dengue poly‐immunoglobulin G scaffold (D‐PIGS) to Fc gamma receptors. (a) Schematic representation of affinity vs avidity measurement. (b) Affinity measurement of the interaction between immobilized (200RU) unfractionated D‐PIGS and hIgG1 to CD64 (120, 60, 30, 15 nm) and CD16a (800, 400, 100, 25, 12.5 nm). The panel on the right indicates calculated affinity constants ( *K*_D_ ) for each interaction as calculated using either the 1 : 1 Langmuir model (CD64) or the ‘two‐state reaction’ model (CD16a). (c) Binding (avidity) of unfractionated D‐PIGS and human IgG1 to his‐tag captured (immobilized) CD64, CD32a and CD16a receptors at a surface density of 200 pg/mm^2^.

We next tested D‐PIGS binding to high‐ and low‐affinity Fc gamma receptors in comparison with human IgG1, by surface plasmon resonance analysis (Biacore). Two assays were performed, as illustrated in Figure 3a. First, increasing concentrations of human CD64 and CD16a were used as analytes to measure the kinetics of the FcR/Fc interaction. Both D‐PIGS and hIgG1 displayed rapid binding kinetics and slow dissociation from the high‐affinity receptor CD64 (Figure 3b), resulting in calculated affinity constants well above 10^9^ (right panel and Table S1). In contrast, when tested for binding to the low‐affinity receptor CD16a, only D‐PIGS displayed prolonged dissociation kinetics in keeping with significant binding, whereas hIgG1 dissociated extremely rapidly, indicating a very low affinity of binding (right‐hand panel). These findings are consistent with previous reports that the removal of core fucose in either the plant or mammalian linkages increases affinity for Fc receptors including CD16a (Forthal *et al*., 2010; Shields *et al*., 2002).

To determine whether D‐PIGS complexes displayed enhanced avidity in respect to FcR binding, we designed a reversed assay layout (Figure 3a, right panel) with the FcR immobilized to the chip via an anti‐his antibody and the unfractionated D‐PIGS complex supplied in the fluid phase (Figure 3c). A receptor density of 200 pg/mm^2^ was chosen to allow multiple interactions between FcRs and Fc domains in the D‐PIGS to occur. Due to the extremely high affinity of CD64 to hIgG1 and to the D‐PIGS (left panel), both samples did not dissociate from the surface for the duration of the injection. In contrast, for both low‐affinity receptors, D‐PIGS remained bound while hIgG1 dissociated within 5 s (CD32a) or at 58 s postinjection (CD16a). We repeated this assay with D‐PIGS material fractioned by HPLC (Figure 3d) and observed that the polymer fraction was retained to CD16a (time to 50% dissociation >600 s), while the M/S fraction was released with a similar decay profile to hIgG1 (time to 50% dissociation 61 and 58 s, respectively). Interaction with CD32a and the high‐affinity receptor CD64 was also markedly stabilized in the polymeric fraction (Tables S1 and S2). These data show that D‐PIGS polymers are capable of establishing long‐lasting interactions with low‐affinity Fc receptors.

### *In vitro* characterization of D‐PIGS

**Figure 4:**
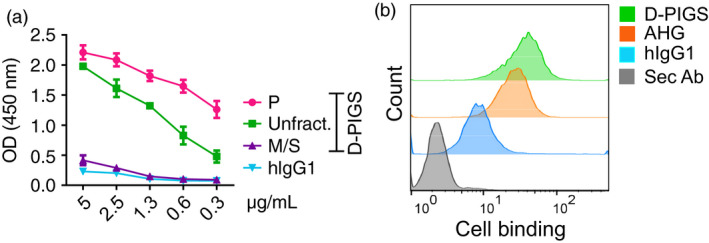
In vitro binding of dengue poly‐immunoglobulin G scaffold (D‐PIGS) to C1q and U937 cells. (a) ELISA showing concentration‐dependent binding of various D‐PIGS fractions to immobilized C1q. Shown are titration curves for twofold serial dilutions of indicated PIGS fractions of monomeric IgG binding to C1q immobilized at 10 μg/mL. (b) Flow cytometry analysis of the binding of D‐PIGS to the surface of U937 human monocyte cells. Cells were incubated with 50 μg/mL of D‐PIGS or human monomeric or heat‐aggregated IgG (AHG) IgG as the negative and positive controls, respectively, for 2 h prior to analysis. Cells stained with the secondary antibody alone were used as the background control. 10 000 cells were analysed.

Dengue PIGS were tested for capacity to bind to C1q component of the complement and to the U937 monocyte cell line expressing the Fc gamma receptors. As shown in Figure 4a, D‐PIGS bound to C1q component of the complement in the concentration‐dependent manner, as would immune complexes do, while monomeric hIgG1 did not. Interestingly, purified polymers bound better than the unfractionated D‐PIGs, while the monomer/single chain fraction showed little to no binding, as expected. This finding confirms the presence of functional polymers in the D‐PIGS preparation and is an indication of their immunogenic potential. Furthermore, D‐PIGS bound efficiently to the surface of U937 cells (used here as a model APC) and this binding was comparable to that of heat‐aggregated human IgG (Figure 4b). Although hIgG1 also showed some binding (due to the presence of CD64 on these cells), this was significantly weaker compared to D‐PIGS. However, in a direct comparison between unfractionated and fractionated D‐PIGS, no significant differences could be observed in binding to U937 cells (Figure S3a). Taken together, these two *in vitro* assays demonstrate the functionality of D‐PIGS and their capacity to interact with the immune cells and molecules.

### IgG response after immunization of CD64 transgenic mice with D‐PIGS

**Figure 5:**
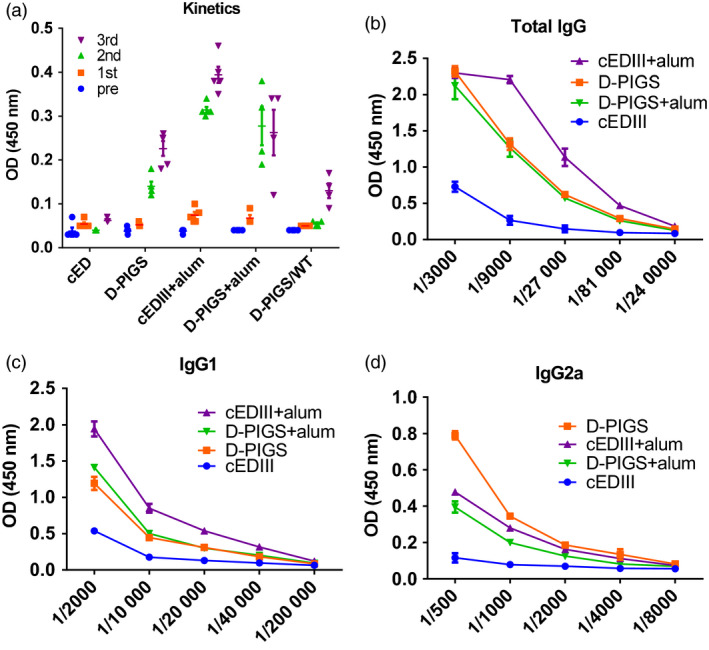
IgG response in sera of CD64 Tg mice immunized with dengue poly‐immunoglobulin G scaffold (D‐PIGS). (a) Kinetics of the cEDIII‐specific IgG response in sera after each immunization; 1 : 1000 serum dilution was used. Shown are the means ± SE for 5 mice per group. (b) Endpoint titres induced by D‐PIGS determined in pooled sera from five mice at equal ratios. (c and d) IgG1 and IgG2a endpoint titres in mice immunized with D‐PIGS (determined as in b).

Having confirmed the functionality of D‐PIGS molecules *in vitro*, we next tested their immunogenicity in mice transgenic for human high‐affinity receptor CD64. Mice were immunized three times in total, and after each immunization, blood samples were taken for analysis of serum IgG responses. As can be seen in Figure 5a, no significant responses were detected after the first immunization for any of the experimental groups. However, after second immunization, IgG was detected in all groups except in mice immunized with cEDIII alone or in wild‐type mice immunized with D‐PIGS. The response was further enhanced after the third immunization, with the groups co‐administered with alum showing the highest responses at the serum dilution used (1 : 1000). Interestingly, D‐PIGS induced a low level response in wild‐type mice, possibly due to cross‐reactivity with mouse Fc receptors or other immune complex capturing mechanisms. We next determined endpoint titres in the immune sera (Figure 5b) and these were 1 : 9000 for cEDIII alone and 1 : 81 000 for D‐PIGS with or without alum. Similar to D‐PIGS, the monomeric form also induced a comparable IgG response in CD64 transgenic mice, signifying the role of the high‐affinity receptor in mediating uptake of these fusion proteins (Figure S3b). We further analysed the relative proportion of IgG1 and IgG2a subtypes in the IgG response, which is an indication of Th2 or Th1 bias. As shown in Figure 5c and d, IgG1 showed significantly higher endpoint titres compared to IgG2a which suggests a possible Th2 bias, although some Th1 response at least is also evident.

### DENV neutralizing capacity of D‐PIGS‐induced antibodies

**Figure 6:**
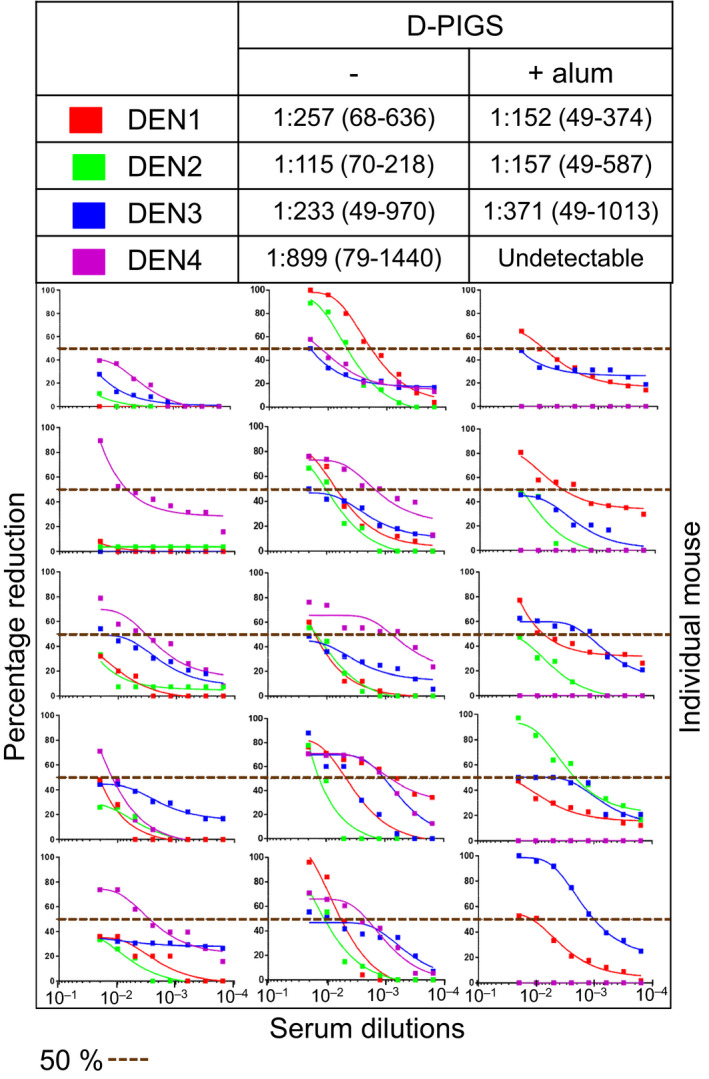
Neutralizing activity of dengue poly‐immunoglobulin G scaffold (D‐PIGS) immune sera from mice. Shown are the focus reduction neutralization test scatter plots and the titration curves from serial dilutions of immune sera from five mice for cEDIII alone and for D‐PIGS, with or without alum. 50% neutralization cut‐off (perforated line) is indicated.

As neutralizing antibodies are an essential component of a protective immune response against dengue infection, we next tested the neutralizing potential of the immune sera against all four serotypes of the dengue virus. This was performed in the standard plaque reduction assay by determining FRNT_50_ values against each serotype. The results are shown in Figure 6. Sera from mice immunized with cEDIII alone showed neutralizing potential only against DENV4 (4/5) while failing to neutralize any other serotypes. Sera from mice immunized with D‐PIGS showed a superior neutralizing potential against all serotypes, although DENV3 was neutralized robustly by serum from one animal only, while other four animals showed borderline neutralizing activity (50% inhibition was achieved only with the first serum dilution of 1 : 50). The titration curves show the range of focus reduction neutralization test (FRNT) titres and serum dilutions. The titres of all the non‐neutralizing mice were scored arbitrarily as 1 : 25 with 50 being the minimum starting serum dilution. Overall, the D‐PIGS‐vaccinated mice exhibited strong neutralizing antibodies titre with mean titre of 1 : 257 (1 : 68–1 : 636) for DENV1, 1 : 115 (1 : 70–1 : 218) for DENV2, 1 : 233 (1 : 50–1 : 970) for DENV3 and 1 : 899 (1 : 79–1 : 1440) for DENV4. In contrast, although mice immunized with D‐PIGS with alum showed comparable antibody titres to D‐PIGS alone, this was not reflected in enhanced neutralizing activity, which in fact was lower. Similarly, addition of alum did not increase DENV neutralizing activity of sera despite increased antibody titres (Figure S4). It appears that the presence of alum somewhat diminished the neutralizing activity of the immune sera, possibly reflecting qualitative differences conferred by the two immunization regimens.

### Cellular immune response to D‐PIGS immunization

**Figure 7:**
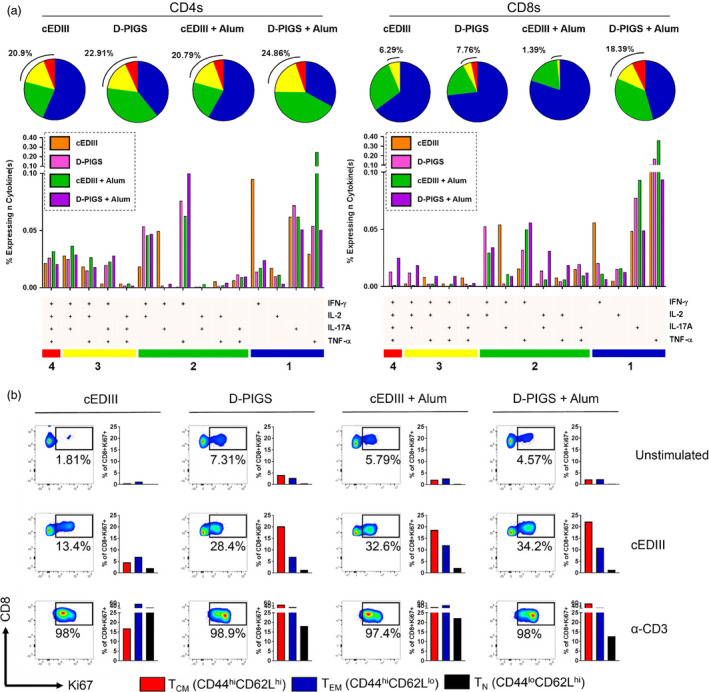
Cellular immune responses induced by dengue poly‐immunoglobulin G scaffold (D‐PIGS). (a) T cells were assessed for polyfunctionality after exposure to recall antigen in the CD4^+^ (left) and CD8^+^ (right) compartments. The gating strategy is shown in Figure S4. Pie charts depict sum total of 1 (blue), 2 (green), 3 (yellow) and 4 cytokine‐positive (red) responses, with values for specific cytokine combinations shown below. (b) Splenocytes were cultured for a further 5 days and assessed for intracellular expression of Ki67 in gated CD8^+^ cells, alongside extracellular levels of CD44 and CD62L. Shown are representative plots of proliferating cells.

We next investigated cellular responses to our vaccine. Emerging evidence suggests that polyfunctional T cells in particular play a protective role in dengue immunization (Yauch *et al*., 2009). Therefore, CD4^+^ and CD8^+^ T cells were assessed for production of combinations of the effector cytokines IFN‐γ, IL‐2, IL‐17A and TNF‐α after antigen recall (gating strategy shown in Figure S5). As can be seen in Figure 7a, cEDIII alone induced 20.9% and 6.29% CD4^+^ and CD8^+^ polyfunctional T cells, respectively. A striking observation was the total lack of quadruple cytokine producers in the CD8 compartment. Interestingly, the monofunctional phenotype in the cEDIII immunization group was defined by a strong IFN‐γ signature. D‐PIGS alone induced 22.91% CD4^+^ and 7.79% CD8^+^ polyfunctional T cells, and this was enhanced even further with adjuvant, leading to an increase of 24.86% CD4^+^ and 18.39% CD8^+^ polyfunctional T cells.

Given the importance of CD8^+^ T cells in protection against DENV—and the observation that D‐PIGS induced quadruple cytokine producing cytotoxic cells—we further probed the CD8^+^ memory T‐cell compartment in response to D‐PIGS immunization. Splenocytes from immunized mice were cultured for several more days in the presence of recall antigen or positive control (recombinant anti‐CD3 antibody) and then assessed for expression of the proliferation marker Ki67. As shown in Figure 7b, there was a dramatic increase in the overall percentage of proliferating cytotoxic cells in splenocytes from mice immunized with D‐PIGS compared to cEDIII antigen alone (13.4% Ki67^+^ vs 28.4% Ki67^+^ cells, respectively). Consistent with the polyfunctionality, this was further boosted in the alum immunization group (34.2% Ki67^+^ cells), although cEDIII adjuvanted with alum showed a comparable increase in proliferation (32.6%). Proliferating cells were then examined for memory T‐cell phenotype, using CD44 and CD62L to denote T central memory (T_CM_: CD44^hi^ CD62L^hi^), T effector memory (T_EM_: CD44^hi^ CD62L^lo^) and naïve T cells (T_N_: CD44^lo^ CD62L^hi^). While there were only minimal differences in the percentages of proliferating T_EM_ cells between immunization groups, there was an approximately fourfold increase in the percentage of proliferating T_CM_ cells in D‐PIGS (±alum) compared to cEDIII alone. Lastly, it was found that enhanced proliferation was associated with a higher quantity of secreted IFN‐γ in the recall culture (Figure S3c). It was therefore concluded that D‐PIGS increased the both the quality and magnitude of the cellular response, as evidenced by increased T‐cell function and proliferative capacity of various memory subsets.

## Discussion

We describe a novel vaccination approach against dengue infection based on Fc‐antigen fusion approach. We previously expressed D‐PIGS molecules based on murine IgG2a and showed that they were highly immunogenic in mice (Kim *et al*., 2017). To translate this promising vaccine approach into a potential dengue vaccine candidate in humans, we generated the human version of D‐PIGS and purified them to a high degree of homogeneity. D‐PIGS were highly immunogenic in transgenic mice expressing human IgG high‐affinity receptor and more importantly in human tonsil cultures. Thus, D‐PIGS induced T‐cell memory responses, IFN‐γ production and neutralizing antibodies against all four DENV serotypes. This nonlive, subunit dengue vaccine based on the consensus EDIII domain and human IgG1 polymeric scaffold has many potential advantages over the other vaccination platforms, including simplicity of production, easy scalability, minimal risk of contamination with animal pathogens, and most importantly, it does not suffer from antigenic interference typically associated with a conventional tetravalent vaccine. The latter advantage is conferred by the use of consensus domain III sequence that is expressed as a single protein fused to human IgG1‐Fc.

The role of EDIII in inducing neutralizing antibodies is somewhat paradoxical, in that human sera from dengue patients appear to contain only a low proportion of such antibodies directed to EDIII (Chen *et al*., 2016; Wahala *et al*., 2009; Williams *et al*., 2012) and yet, there are a number of strongly neutralizing monoclonal antibodies reported (Crill and Roehrig, 2001; Roehrig *et al*., 1998; Shrestha *et al*., 2010; Sukupolvi‐Petty *et al*., 2007). While most of these monoclonal antibodies target linear epitopes, some of the strongly neutralizing mAbs such as 2D22 target a quaternary epitope on EDIII (Gallichotte *et al*., 2015). Similarly, rabbit antisera targeted to EDIII was strongly neutralizing and induced very little ADE, suggesting that EDIII may be used as a subunit vaccine to induce an effective and safe antibody response. This is consistent with the concept of unnatural immunity, whereby natural infection does not seem to provoke neutralizing anti‐EDIII response while increasing the risk of cross‐reactivity and ADE, yet vaccine‐induced EDIII antibodies may confer protection. Indeed, a study of human antidengue neutralizing antibodies showed that they predominantly recognize complex epitopes present on the intact virions but not recombinant envelope ectodomain protein (de Alwis *et al*., 2012). In this study, we used a consensus domain III (Leng *et al*., 2009) obtained by alignment of amino acid sequences from different isolates of the four serotypes of dengue viruses, rather than the mixture of four individual serotype‐specific domains, thus simplifying significantly the vaccine formulation.

Dengue PIGS induced strong antibody responses in transgenic mice expressing human IgG high‐affinity receptor (CD64). Although both IgG1 and IgG2a were present, a much higher titre of IgG1 was detected, suggesting a Th2 bias. Both monomeric and polymeric D‐PIGS were immunogenic in mice (Figure S3b). This is in contrast with human immune response in tonsil cultures which indicated polymer superiority in inducing an IgG response and could be explained by only partial suitability of the transgenic CD64 mouse model. As both monomer and polymer can bind to high‐affinity IgG receptor but only polymer binds to the low‐affinity receptors, the single receptor transgene model may not be sufficiently discriminatory, unlike the human tissue, which displays all the IgG receptors. Nevertheless, the induced mouse cEDIII‐specific antibodies were able to neutralize dengue serotypes 1, 2 and 4 with similar efficiency, with only DENV3 showing lower FRNT values. Interestingly, despite inducing higher antibody titres to D‐PIGS, addition of alum as the adjuvant did not enhance neutralization potential of induced antibodies and in fact, appeared to have a detrimental effect. Although we have no experimental evidence, we speculate that this is most likely due to qualitative differences in the functionality of the induced antibodies.

The importance of T‐cell responses in dengue infection has been very much in focus recently. While effector T cells are partially responsible for the immunopathology of dengue disease, effective vaccine‐induced protection depends on these cells. This was elegantly demonstrated in experiments by Yauch *et al*. (2009) who showed that depletion of CD4^+^ T cells during natural infection had no effect on viral control, whereas depletion of this subset during DENV peptide immunization led to a total loss of vaccine‐induced protection. Dengue induces a number of CD4^+^ T‐ and CD8^+^ T‐cell epitopes to both structural and nonstructural proteins recognized in mice (Rothman *et al*., 1996) and humans (Weiskopf *et al*., 2015b,2015c). CD8^+^ T‐cell responses (particularly of a polyfunctional phenotype) are generally thought to play an important role in controlling infection and thus the severity of the disease, partly supported by experimental evidence (Gil *et al*., 2009, 2014; Yauch *et al*., 2009). Although nonstructural proteins are the dominant targets of the CD8^+^ T‐cell responses (Weiskopf *et al*., 2013), some CD8^+^ T‐cell epitopes are present in DIII of the envelope protein (Chiang *et al*., 2016; Weiskopf *et al*., 2015a). In our studies, we detected cEDIII‐specific both CD4^+^ and CD8^+^ T‐cell proliferation in tonsillar cell populations stimulated *in vitro*. This proliferative response was associated with IFN‐γ production. Interestingly, polymeric D‐PIGS were more efficient in inducing these cellular responses than the monomeric version, most probably due to a more efficient binding of the Fc receptors, as demonstrated in our *in vitro* Biacore assays. In our *in vivo* experiments, D‐PIGS were more adept than single antigen at producing proliferative T_EM_ and T_CM_ responses, alongside increased IFN‐γ production and a greater degree of polyfunctionality. T_CM_ are of particular interest as these memory cells can serve as a long‐lasting reservoir of immunological memory with high proliferative capacity. Together, these human and mouse cellular data underscore the immunological superiority of D‐PIGS over single antigen immunization.

The advantages of plants as an expression system have been well documented. One of the key advantages is the capacity for almost enormous scale‐up production of pharmaceutical proteins which may be required to meet ever increasing global demand. Existing microbial systems simply cannot meet that demand because they are either very expensive, or the proteins of interest, such as antibodies and the Fc‐fusion proteins described here, are too complex to be made in such systems. Animal or human cell cultures provide an alternative, but are expensive. Like animals, plants are complex, multicellular organisms and their protein synthesis is more similar to that of animals than those of bacteria or other bio‐microorganisms. To further reduce differences in post‐translational modification of plant‐expressed proteins, various genetic plant mutants have been generated, including the delta XF tobacco line used in this study. Glyco‐engineering in plant is developed for pharmaceutical products and well documented (Forthal *et al*., 2010; Strasser, 2016). Thus, Strasser *et al*. (2004) produced a triple knockout in *Arabidopsis* that downregulates β‐(1,2) XylT and α‐(1,3) FucT activity, resulting in the absence of plant‐specific glycans which are occasionally involved in inducing glycan‐specific IgE (Reusch and Tejada, 2015), and a product that is much more similar to human glycoproteins. D‐PIGS without plant‐specific fucose and xylose show higher affinity for FcγRIII than D‐PIGS from wild‐type *N. benthamiana*, and this is consistent with the findings from another study reporting enhanced binding to human FcγRIII and antibody‐dependent cellular toxicity on glyco‐engineered human IgG1 (Shields *et al*., 2002). The D‐PIGS molecules produced in delta XF plants as described here could therefore be feasibly produced and purified to a high degree of purity and homogeneity to meet the good practice manufacturing requirements for safe human applications.

In conclusion, we described a novel plant‐expressed subunit vaccine candidate for dengue vaccination based on Fc‐antigen fusion protein approach that could potentially be used as a boost to a viral‐based dengue vaccine. Humanized D‐PIGS show a desirable immunogenic profile in mice transgenic for human IgG Fc receptor and in human tonsil tissues and importantly, induce neutralizing antibody responses and cellular immunity, which are both required in a protective antidengue response. This novel dengue vaccine candidate merits further evaluation as a boost to Dengvaxia or DENVax in the nonhuman primate model of dengue infection, to achieve complete protection against all for DENV virus serotypes.

## Materials and methods

### Plant expression, detection, purification and fractionation of D‐PIGS

Vector construction for D‐PIGS, agro‐infiltration of *N. benthamiana* plant leaves, purification and detection by SDS‐PAGE and Western blotting, as well as fractionation of low and high molecular weight forms, were all described in detail previously (Kim *et al*., 2017). Briefly, the agrobacteria (GV3101 strain) transformed with D‐PIGS constructs and human J chain (co‐expression to facilitate the assembly of PIGS into polymers) were cultured at 28 °C in YENB medium containing antibiotics (50 μg/mL each of carbenicillin, kanamycin and rifampicin) for 2 days. Cells were collected by centrifugation and infiltrated into tobacco leaves. Leaf extracts were prepared 7 days later and proteins separated on a 4%–12% Bis‐Tris SDS‐PAGE gel (Thermo Fisher, Paisley, UK), followed by Coomassie Blue staining or blotting onto a nitrocellulose membrane. Blots were probed with peroxidase‐conjugated anti‐human IgG antiserum (1 : 1000 dilution; The Binding Site Birmingham, UK) for detection of the IgG Fc portion, or with antidengue virus monoclonal antibody (1 : 2500 dilution; Bio‐Rad AbD Serotec Kidlington, UK) followed by anti‐mouse IgG peroxidase‐conjugated antiserum (1 : 1000 dilution; The Binding Site). For purification of D‐PIGS, standard protein A chromatography procedure was applied, followed by concentrating the protein by ultrafiltration (Centricon, Merck‐Millipore, Watford, UK) to 1 mg/mL. For some experiments, purified D‐PIGS were fractionated by HPLC on a Shimadzu LC2010AHT system (Milton Keynes, UK). Separation was achieved in phosphate‐buffered saline (PBS) at a flow rate of 1 mL/min. As reference, the Bio‐Rad GFC Standards were used.

### FcγR binding kinetics

The affinity and avidity of the D‐PIGS complexes in binding to human Fcγ receptors were measured using a Biacore X100 instrument (GE Life Sciences, Little Chalfont, UK). For the affinity measurements, 5000 relative units (RU) of a protein A (P6032, Merck, Poole, UK) in pH 5.5 acetate buffer (GE Life Sciences) was immobilized on both flow channels of a CM5 sensor chip using amine coupling chemistry (His capture kit; GE Life Sciences). For each cycle, 200 RU of the sample (PIGS or hIgG1) was captured on flow channel 2. The analyte, either recombinant human CD16a (4325‐FC; R&D Systems, Abingdon, UK) or recombinant human CD64 (1257‐FC‐050; R&D Systems), was injected in a concentration series from 12.5 to 800 nm over both flow channels with a contact time of 60 s and a flow rate of 50 μL/min and dissociation monitored for 200 s. Regeneration of the surface was achieved by two 30‐s pulses of 10 mm glycine pH 1.5.

Data were fitted to the predefined ‘two‐state interaction’ model for CD16a (Heider *et al*., 2011)and the ‘1 : 1 interaction’ model for CD64.

For the avidity assay, 12 000 RU of an anti‐his antibody was immobilized on both flow channels of a CM5 sensor chip using amine coupling chemistry (His capture kit; GE Life Sciences). For each sample, recombinant human CD16 or CD64 (as above) diluted to 2 μg/mL in HBS‐EP+ running buffer was captured on flow channel 2 to a level of 200 RU. Samples (1 μm, 500 nm and 166.7 nm for CD64, CD32a and CD16a, respectively) were injected over both flow channels with a contact time of 80 s and a flow rate of 30 μL/min, and dissociation was monitored for at least 500 s. Regeneration of the surface was achieved by a 30‐s pulse of 10 mm glycine pH 1.5.

### Immunization of mice

For immunization with D‐PIGS, 12‐ to 20‐week‐old inbred male and female FcγRI/CD64 mice were used. These mice were originated in 1996 (Heijnen *et al*., 1996) and a colony is kept and bred at St George's since 2010 under the establishment licence. The mice were kept under defined environmental conditions, and all experimental work was approved by St George's Ethics Committee and the UK Home Office. Five mice per group (both males and females, aged 8–20 weeks) were immunized subcutaneously in the base of tail with 25 μg of D‐PIGS in 100 μL, with or without aluminium hydroxide gel (Sigma). Control mice were immunized with saline solution or with 6 μg cEDIII alone (equivalent amount of the antigen within D‐PIGS). Mice were immunized two more times, at weeks 2 and 4, and were bled after each immunization to monitor the antibody titres. At week 6, mice were sacrificed and bled by cardiac puncture and the spleens were collected for analysis of T‐cell responses.

### Focus reduction neutralization test

Neutralization capacity of sera samples against dengue 1–4 (DENV1 West Pac/74—Nauru 1974; DENV2 S‐16803—Thailand 1974; DENV3 CH 53489—Thailand 1973; DENV4 TVP‐360/S341750—Columbia 1982) was determined by FRNT on Vero cells (ATCC) seeded in 96‐well flat‐bottom plates at a density of 20 000 cells per well in DMEM supplemented with 10% FBS. Cells were cultured for 24 h before infection. Sera were thawed and heat‐inactivated at 56 °C for 30 min and serially diluted from 1 : 25 to 1 : 3200 in 100 μL Opti‐MEM (Thermo Fisher, Hemel Hempstead, UK) serum‐free medium and an equal volume of dengue virus containing 200 PFU was added and incubated for 1 h at 37 °C in 96‐well U‐bottom cell culture plates. Vero monolayer was then infected with 50 μL of the serum–virus mixture and further incubated for 1 h at 37 °C making the final serum dilution to 1 : 50 in the first well and final PFU to 100. The cells were incubated at 37 °C for 3 days with an overlay of 2% methylcellulose (Sigma) in Opti‐MEM containing 20 μg/mL of ciprofloxacin (Sigma) and 2.5 μg/mL of amphotericin B (Himedia, Mumbai, India). At termination, cells were fixed and stained with anti‐Flavivirus Group Antigen Antibody, clone D1‐4G2‐4‐15 (Merck‐Millipore, Watford, UK) at 1 : 2500 dilution followed by HRP‐linked anti‐mouse IgG (Cell Signaling Danvers, MA). Foci were developed with TrueBlue Peroxidase (KLP). Separate plates were used for each serotype to account for different time intervals required for colour development. FRNT_50_ was calculated where 50% reduction in foci was observed as compared to control wells (virus only).

### Intracellular cytokine staining and flow cytometry

T‐cell polyfunctionality was measured as previously described (Kim *et al*., 2017). Briefly, splenocytes were stimulated with 5 μg/mL cEDIII for 4 h in the presence of 5 μg/mL brefeldin A. Cells were then washed with PBS and stained with a viability dye (eFluor 780; 1:1000 dilution; Thermo Fisher, Hemel Hempstead, UK) alongside an Fc receptor blockade (TruStain, 1 μg/mL; Biolegend London, UK) for 20 min at 4 °C. Following this, cells were washed in flow cytometry buffer and then fixed in 100 μL BD Cytofix (Becton Dickinson, Oxford, UK) for 30 min at 4 °C. Cells were washed and then stained with the following antibodies at optimized concentrations for 45 min at 4 °C: CD3‐FITC, CD4‐PerCP‐Cy5.5, CD8‐Alexa Fluor 700, IFN‐γ‐PE Dazzle, IL‐2‐PE, IL‐17A‐PE‐Cy7 and TNF‐α‐APC (all from Biolegend). Fluorescence minus one and PMA/ionomycin‐stimulated cells were used to determine gating boundaries and serve as positive controls. Cells were then washed twice with permeabilization buffer and flow cytometry buffer and then acquired on a LSR II (Beckton Dickinson, Oxford, UK) instrument.

### Immune responses to D‐PIGS in human adenoid–tonsillar tissue culture *ex vivo*

Adenoids and palatine tonsils were obtained from patients (aged 3–30 years) who underwent the adenoidectomy and/or tonsillectomy due to upper airway obstruction at Liverpool Alder Hey Children's Hospital and Royal Liverpool and Broadgreen University Hospitals (REC approval reference: 14/SS/1058). Written informed consent was obtained from each patient. Adenoid–tonsillar mononuclear cells (MNCs) were isolated following Ficoll gradient centrifugation. For detection of T‐cell proliferative responses, the MNCs were stained with carboxyfluorescein succinimidyl ester (CFSE), followed by cell stimulation with the D‐PIGS (25 μg/mL) or with cEDIII antigen alone (5 μg/mL). At day 3, cell culture supernatants were collected for IFN‐γ analysis by ELISA. At day 5, flow cytometry was performed to analyse CD4^+^ and CD8^+^ T‐cell proliferative responses using CFSE (5(6)‐carboxyfluorescein *N*‐hydroxysuccinimidyl ester) cell tracing (Zhang *et al*., 2007). Lymphocyte population was first gated using typical forward and side scatter properties as indicated in Figure S6 (which typically gave a viability greater >95% confirmed by propidium iodide staining). Singlet population was gated and followed by sequential staining for CD3/CD4/CD8/IFN‐γ as shown. Flowjo software was used for flow data analysis. For detection of B‐cell antibody production, tonsillar MNCs were stimulated by the vaccines or antigens for up to 2 weeks. Cell culture supernatants were harvested and analysed by a standard ELISA procedure as described previously (Zhang *et al*., 2006) for cEDIII antigen‐specific IgG antibody responses.

### Additional methods

Binding of D‐PIGS to U937 monocytic cells and C1q in ELISA (Appendix S1), humoral responses analysis in sera (Appendix S2) and T‐cell proliferation assay and IFN‐γ measurement (Appendix S3) are described in supportive information file.

### Statistical analysis

The cell culture‐based assays were performed in triplicate, and the values (from a representative experiment of typically 3 performed) are shown as the mean ± standard deviation. For immunization experiments, five animals were used per group (in two independent experiments). For all assays which had more than two experimental variables, Dunnett's multiple comparison test was used. GraphPad Prism v.7 (GraphPad Software Inc., La Jolla, CA) software was used for statistical analysis, and the differences were significant when the *P* value was 0.05 or less.

## Conflict of interest

The authors declare no conflict of interest.

## Author contributions

MYK, JM and RR conceived and developed the work plan and co‐wrote the manuscript, with MYK also performing most of the experimental work. AC performed intracellular cytokine staining; GRD performed T‐cell proliferation assays; KN and AC performed dengue neutralization assays; MSA and QZ performed human tonsil culture assays; SH performed HPLC analysis; MJP performed Biacore assays, and MSY and YSJ provided critical input to assessment of the data and financial support through research grants.

## Supporting information


**Figure S1** Comparative analysis of D‐PIGS expressed in wild type and ∆XF Benthamiana plants.
**Figure S2** Temperature stability of high molecular weight D‐PIGS.
**Figure S3** Comparative analysis of low and high molecular weight D‐PIGS.
**Figure S4** Dengue virus Neutralization curves obtained with sera from mice immunised with cEDIII antigen alone or in combination with Alum.
**Figure S5** Gating strategy for analysing T‐cell intracellular cytokine staining by flow cytometry.
**Figure S6** Gating strategy for analysing tonsillar T‐cell proliferative response by CFSE staining and flow cytometry.
**Table S1** Kinetics data for D‐PIGS interactions with IgG Fc receptors by surface plasmon resonance.
**Table S2** Time to 50% dissociation of antibody analyte from receptor ligand.
**Appendix S1** Functional characterisation of D‐PIGS by C1q ELISA and cell surface binding; protocol description.
**Appendix S2** Humoral responses in sera of immunized mice; protocol description.
**Appendix S3** T‐cell proliferation and IFN‐γ; protocol description.

## References

[pbi12869-bib-0001] de Alwis, R. , Smith, S.A. , Olivarez, N.P. , Messer, W.B. , Huynh, J.P. , Wahala, W.M. , White, L.J. *et al*. (2012) Identification of human neutralizing antibodies that bind to complex epitopes on dengue virions. Proc. Natl Acad. Sci. USA, 109, 7439–7444. 22499787 10.1073/pnas.1200566109 PMC3358852

[pbi12869-bib-0002] Arntzen, C. (2015) Plant‐made pharmaceuticals: from ‘Edible Vaccines’ to Ebola therapeutics. Plant Biotechnol. J. 13, 1013–1016. 26345276 10.1111/pbi.12460 PMC5049623

[pbi12869-bib-0003] Bhatt, S. , Gething, P.W. , Brady, O.J. , Messina, J.P. , Farlow, A.W. , Moyes, C.L. , Drake, J.M. *et al*. (2013) The global distribution and burden of dengue. Nature, 496, 504–507. 23563266 10.1038/nature12060 PMC3651993

[pbi12869-bib-0004] Brewoo, J.N. , Kinney, R.M. , Powell, T.D. , Arguello, J.J. , Silengo, S.J. , Partidos, C.D. , Huang, C.Y. *et al*. (2012) Immunogenicity and efficacy of chimeric dengue vaccine (DENVax) formulations in interferon‐deficient AG129 mice. Vaccine, 30, 1513–1520. 22178727 10.1016/j.vaccine.2011.11.072 PMC4592107

[pbi12869-bib-0005] Capeding, M.R. , Tran, N.H. , Hadinegoro, S.R. , Ismail, H.I. , Chotpitayasunondh, T. , Chua, M.N. , Luong, C.Q. *et al*. (2014) Clinical efficacy and safety of a novel tetravalent dengue vaccine in healthy children in Asia: a phase 3, randomised, observer‐masked, placebo‐controlled trial. Lancet, 384, 1358–1365. 25018116 10.1016/S0140-6736(14)61060-6

[pbi12869-bib-0006] Chen, J. , Wen, K. , Li, X.Q. , Yi, H.S. , Ding, X.X. , Huang, Y.F. , Pan, Y.X. *et al*. (2016) Functional properties of DENV EDIII‐reactive antibodies in human DENV1‐infected sera and rabbit antiserum to EDIII. Mol. Med. Rep. 14, 1799–1808. 27357403 10.3892/mmr.2016.5454

[pbi12869-bib-0007] Chiang, C.Y. , Pan, C.H. , Chen, M.Y. , Hsieh, C.H. , Tsai, J.P. , Liu, H.H. , Liu, S.J. *et al*. (2016) Immunogenicity of a novel tetravalent vaccine formulation with four recombinant lipidated dengue envelope protein domain IIIs in mice. Sci. Rep. 6, 30648. 27470096 10.1038/srep30648 PMC4965760

[pbi12869-bib-0008] Crill, W.D. and Roehrig, J.T. (2001) Monoclonal antibodies that bind to domain III of dengue virus E glycoprotein are the most efficient blockers of virus adsorption to Vero cells. J. Virol. 75, 7769–7773. 11462053 10.1128/JVI.75.16.7769-7773.2001 PMC115016

[pbi12869-bib-0009] Czajkowsky, D.M. , Hu, J. , Shao, Z. and Pleass, R.J. (2012) Fc‐fusion proteins: new developments and future perspectives. EMBO Mol. Med. 4, 1015–1028. 22837174 10.1002/emmm.201201379 PMC3491832

[pbi12869-bib-0010] Deen, J. , Weber, M.W. and Jaenisch, T. (2016) Dengue in the context of the integrated management of childhood illness. PLoS Negl. Trop. Dis. 10, e0004838. 27559725 10.1371/journal.pntd.0004838 PMC4999212

[pbi12869-bib-0011] Durbin, A.P. , Whitehead, S.S. , Shaffer, D. , Elwood, D. , Wanionek, K. , Thumar, B. , Blaney, J.E. *et al*. (2011) A single dose of the DENV‐1 candidate vaccine rDEN1Delta30 is strongly immunogenic and induces resistance to a second dose in a randomized trial. PLoS Negl. Trop. Dis. 5, e1267. 21829748 10.1371/journal.pntd.0001267 PMC3149013

[pbi12869-bib-0012] Forthal, D.N. , Gach, J.S. , Landucci, G. , Jez, J. , Strasser, R. , Kunert, R. and Steinkellner, H. (2010) Fc‐glycosylation influences Fcgamma receptor binding and cell‐mediated anti‐HIV activity of monoclonal antibody 2G12. J. Immunol. 185, 6876–6882. 21041724 10.4049/jimmunol.1002600

[pbi12869-bib-0013] Gallichotte, E.N. , Widman, D.G. , Yount, B.L. , Wahala, W.M. , Durbin, A. , Whitehead, S. , Sariol, C.A. *et al*. (2015) A new quaternary structure epitope on dengue virus serotype 2 is the target of durable type‐specific neutralizing antibodies. mBio, 6, e01461–01415. 26463165 10.1128/mBio.01461-15 PMC4620467

[pbi12869-bib-0014] Gil, L. , Lopez, C. , Blanco, A. , Lazo, L. , Martin, J. , Valdes, I. , Romero, Y. *et al*. (2009) The cellular immune response plays an important role in protecting against dengue virus in the mouse encephalitis model. Viral Immunol. 22, 23–30. 19210225 10.1089/vim.2008.0063

[pbi12869-bib-0015] Gil, L. , Izquierdo, A. , Lazo, L. , Valdes, I. , Ambala, P. , Ochola, L. , Marcos, E. *et al*. (2014) Capsid protein: evidences about the partial protective role of neutralizing antibody‐independent immunity against dengue in monkeys. Virology, 456–457, 70–76. 10.1016/j.virol.2014.03.011 24889226

[pbi12869-bib-0016] Godoi, I.P. , Santos, A.S. , Reis, E.A. , Lemos, L.L. , Brandao, C.M. , Alvares, J. , Acurcio, F.A. *et al*. (2017) Consumer willingness to pay for dengue vaccine (CYD‐TDV, Dengvaxia(R)) in Brazil; implications for future pricing considerations. Front. Pharmacol. 8, 41. 28210223 10.3389/fphar.2017.00041 PMC5288336

[pbi12869-bib-0017] Hadinegoro, S.R. , Arredondo‐Garcia, J.L. , Capeding, M.R. , Deseda, C. , Chotpitayasunondh, T. , Dietze, R. , Muhammad Ismail, H.I. *et al*. (2015) Efficacy and long‐term safety of a dengue vaccine in regions of endemic disease. N. Engl. J. Med. 373, 1195–1206. 26214039 10.1056/NEJMoa1506223

[pbi12869-bib-0018] Harapan, H. , Anwar, S. , Bustamam, A. , Radiansyah, A. , Angraini, P. , Fasli, R. , Salwiyadi, S. *et al*. (2017) Willingness to pay for a dengue vaccine and its associated determinants in Indonesia: a community‐based, cross‐sectional survey in Aceh. Acta Trop. 166, 249–256. 27908746 10.1016/j.actatropica.2016.11.035

[pbi12869-bib-0019] Heider, K.H. , Kiefer, K. , Zenz, T. , Volden, M. , Stilgenbauer, S. , Ostermann, E. , Baum, A. *et al*. (2011) A novel Fc‐engineered monoclonal antibody to CD37 with enhanced ADCC and high proapoptotic activity for treatment of B‐cell malignancies. Blood, 118, 4159–4168. 21795744 10.1182/blood-2011-04-351932 PMC3204733

[pbi12869-bib-0020] Heijnen, I.A. , van Vugt, M.J. , Fanger, N.A. , Graziano, R.F. , de Wit, T.P. , Hofhuis, F.M. , Guyre, P.M. *et al*. (1996) Antigen targeting to myeloid‐specific human Fc gamma RI/CD64 triggers enhanced antibody responses in transgenic mice. J. Clin. Investig. 97, 331–338. 8567952 10.1172/JCI118420 PMC507022

[pbi12869-bib-0021] Kim, M.Y. , Reljic, R. , Kilbourne, J. , Ceballos‐Olvera, I. , Yang, M.S. , Reyes‐del Valle, J. and Mason, H.S. (2015) Novel vaccination approach for dengue infection based on recombinant immune complex universal platform. Vaccine, 33, 1830–1838. 25728317 10.1016/j.vaccine.2015.02.036

[pbi12869-bib-0022] Kim, M.Y. , Kim, B.Y. , Oh, S.M. , Reljic, R. , Jang, Y.S. and Yang, M.S. (2016) Oral immunisation of mice with transgenic rice calli expressing cholera toxin B subunit fused to consensus dengue cEDIII antigen induces antibodies to all four dengue serotypes. Plant Mol. Biol. 92, 347–356. 27566485 10.1007/s11103-016-0517-0

[pbi12869-bib-0023] Kim, M.Y. , Van Dolleweerd, C. , Copland, A. , Paul, M.J. , Hofmann, S. , Webster, G.R. , Julik, E. *et al*. (2017) Molecular engineering and plant expression of an immunoglobulin heavy chain scaffold for delivery of a dengue vaccine candidate. Plant Biotechnol. J. 15, 1590–1601. 28421694 10.1111/pbi.12741 PMC5698049

[pbi12869-bib-0024] Leng, C.H. , Liu, S.J. , Tsai, J.P. , Li, Y.S. , Chen, M.Y. , Liu, H.H. , Lien, S.P. *et al*. (2009) A novel dengue vaccine candidate that induces cross‐neutralizing antibodies and memory immunity. Microbes Infect. 11, 288–295. 19114121 10.1016/j.micinf.2008.12.004

[pbi12869-bib-0025] Lyon, G.M. , Mehta, A.K. , Varkey, J.B. , Brantly, K. , Plyler, L. , McElroy, A.K. , Kraft, C.S. *et al*. (2014) Clinical care of two patients with Ebola virus disease in the United States. N. Engl. J. Med. 371, 2402–2409. 25390460 10.1056/NEJMoa1409838

[pbi12869-bib-0026] Mekhaiel, D.N. , Czajkowsky, D.M. , Andersen, J.T. , Shi, J. , El‐Faham, M. , Doenhoff, M. , McIntosh, R.S. *et al*. (2011) Polymeric human Fc‐fusion proteins with modified effector functions. Sci. Rep. 1, 124. 22355641 10.1038/srep00124 PMC3216605

[pbi12869-bib-0027] Osorio, J.E. , Huang, C.Y. , Kinney, R.M. and Stinchcomb, D.T. (2011) Development of DENVax: a chimeric dengue‐2 PDK‐53‐based tetravalent vaccine for protection against dengue fever. Vaccine, 29, 7251–7260. 21777638 10.1016/j.vaccine.2011.07.020 PMC4592106

[pbi12869-bib-0028] Poggianella, M. , Slon Campos, J.L. , Chan, K.R. , Tan, H.C. , Bestagno, M. , Ooi, E.E. and Burrone, O.R. (2015) Dengue E protein domain III‐based DNA immunisation induces strong antibody responses to all four viral serotypes. PLoS Negl. Trop. Dis. 9, e0003947. 26218926 10.1371/journal.pntd.0003947 PMC4517776

[pbi12869-bib-0029] Porter, K.R. and Raviprakash, K. (2015) Nucleic acid (DNA) immunization as a platform for dengue vaccine development. Vaccine, 33, 7135–7140. 26458805 10.1016/j.vaccine.2015.09.102

[pbi12869-bib-0030] Reusch, D. and Tejada, M.L. (2015) Fc glycans of therapeutic antibodies as critical quality attributes. Glycobiology, 25, 1325–1334. 26263923 10.1093/glycob/cwv065 PMC4634315

[pbi12869-bib-0031] Roehrig, J.T. , Bolin, R.A. and Kelly, R.G. (1998) Monoclonal antibody mapping of the envelope glycoprotein of the dengue 2 virus, Jamaica. Virology, 246, 317–328. 9657950 10.1006/viro.1998.9200

[pbi12869-bib-0032] Rothman, A.L. , Kurane, I. and Ennis, F.A. (1996) Multiple specificities in the murine CD4^+^ and CD8^+^ T‐cell response to dengue virus. J. Virol. 70, 6540–6546. 8794288 10.1128/jvi.70.10.6540-6546.1996 PMC190694

[pbi12869-bib-0033] Saez‐Llorens, X. , Tricou, V. , Yu, D. , Rivera, L. , Tuboi, S. , Garbes, P. , Borkowski, A. *et al*. (2017) Safety and immunogenicity of one versus two doses of Takeda's tetravalent dengue vaccine in children in Asia and Latin America: interim results from a phase 2, randomised, placebo‐controlled study. Lancet Infect. Dis. 17, 615–625. 28365225 10.1016/S1473-3099(17)30166-4

[pbi12869-bib-0034] Shafie, A.A. , Yeo, H.Y. , Coudeville, L. , Steinberg, L. , Gill, B.S. , Jahis, R. and Amar‐Singh, H. (2017) The potential cost effectiveness of different dengue vaccination programmes in Malaysia: a value‐based pricing assessment using dynamic transmission mathematical modelling. Pharmacoeconomics, 35, 575–589. 28205150 10.1007/s40273-017-0487-3

[pbi12869-bib-0035] Shields, R.L. , Lai, J. , Keck, R. , O'Connell, L.Y. , Hong, K. , Meng, Y.G. , Weikert, S.H. *et al*. (2002) Lack of fucose on human IgG1 N‐linked oligosaccharide improves binding to human Fcgamma RIII and antibody‐dependent cellular toxicity. J. Biol. Chem. 277, 26733–26740. 11986321 10.1074/jbc.M202069200

[pbi12869-bib-0036] Shrestha, B. , Brien, J.D. , Sukupolvi‐Petty, S. , Austin, S.K. , Edeling, M.A. , Kim, T. , O'Brien, K.M. *et al*. (2010) The development of therapeutic antibodies that neutralize homologous and heterologous genotypes of dengue virus type 1. PLoS Pathog. 6, e1000823. 20369024 10.1371/journal.ppat.1000823 PMC2848552

[pbi12869-bib-0037] Strasser, R. (2016) Plant protein glycosylation. Glycobiology, 26, 926–939. 26911286 10.1093/glycob/cww023 PMC5045529

[pbi12869-bib-0038] Strasser, R. , Altmann, F. , Mach, L. , Glossl, J. and Steinkellner, H. (2004) Generation of Arabidopsis thaliana plants with complex N‐glycans lacking beta1,2‐linked xylose and core alpha1,3‐linked fucose. FEBS Lett. 561, 132–136. 15013764 10.1016/S0014-5793(04)00150-4

[pbi12869-bib-0039] Sukupolvi‐Petty, S. , Austin, S.K. , Purtha, W.E. , Oliphant, T. , Nybakken, G.E. , Schlesinger, J.J. , Roehrig, J.T. *et al*. (2007) Type‐ and subcomplex‐specific neutralizing antibodies against domain III of dengue virus type 2 envelope protein recognize adjacent epitopes. J. Virol. 81, 12816–12826. 17881453 10.1128/JVI.00432-07 PMC2169112

[pbi12869-bib-0040] Swaminathan, G. , Thoryk, E.A. , Cox, K.S. , Smith, J.S. , Wolf, J.J. , Gindy, M.E. , Casimiro, D.R. *et al*. (2016) A tetravalent sub‐unit dengue vaccine formulated with ionizable cationic lipid nanoparticle induces significant immune responses in rodents and non‐human primates. Sci. Rep. 6, 34215. 27703172 10.1038/srep34215 PMC5050434

[pbi12869-bib-0041] Wahala, W.M. , Kraus, A.A. , Haymore, L.B. , Accavitti‐Loper, M.A. and de Silva, A.M. (2009) Dengue virus neutralization by human immune sera: role of envelope protein domain III‐reactive antibody. Virology, 392, 103–113. 19631955 10.1016/j.virol.2009.06.037 PMC2746956

[pbi12869-bib-0042] Watts, D.M. , Callahan, J. , Rossi, C. , Oberste, M.S. , Roehrig, J.T. , Wooster, M.T. , Smith, J.F. *et al*. (1998) Venezuelan equine encephalitis febrile cases among humans in the Peruvian Amazon River region. Am. J. Trop. Med. Hyg. 58, 35–40. 9452289 10.4269/ajtmh.1998.58.35

[pbi12869-bib-0043] Weiskopf, D. , Angelo, M.A. , de Azeredo, E.L. , Sidney, J. , Greenbaum, J.A. , Fernando, A.N. , Broadwater, A. *et al*. (2013) Comprehensive analysis of dengue virus‐specific responses supports an HLA‐linked protective role for CD8^+^ T cells. Proc. Natl Acad. Sci. USA, 110, E2046–E2053. 23580623 10.1073/pnas.1305227110 PMC3670335

[pbi12869-bib-0044] Weiskopf, D. , Angelo, M.A. , Bangs, D.J. , Sidney, J. , Paul, S. , Peters, B. , de Silva, A.D. *et al*. (2015a) The human CD8^+^ T cell responses induced by a live attenuated tetravalent dengue vaccine are directed against highly conserved epitopes. J. Virol. 89, 120–128. 25320311 10.1128/JVI.02129-14 PMC4301095

[pbi12869-bib-0045] Weiskopf, D. , Bangs, D.J. , Sidney, J. , Kolla, R.V. , De Silva, A.D. , de Silva, A.M. , Crotty, S. *et al*. (2015b) Dengue virus infection elicits highly polarized CX3CR1^+^ cytotoxic CD4^+^ T cells associated with protective immunity. Proc. Natl Acad. Sci. USA, 112, E4256–E4263. 26195744 10.1073/pnas.1505956112 PMC4534238

[pbi12869-bib-0046] Weiskopf, D. , Cerpas, C. , Angelo, M.A. , Bangs, D.J. , Sidney, J. , Paul, S. , Peters, B. *et al*. (2015c) Human CD8^+^ T‐cell responses against the 4 dengue virus serotypes are associated with distinct patterns of protein targets. J. Infect. Dis. 212, 1743–1751. 25980035 10.1093/infdis/jiv289 PMC4633759

[pbi12869-bib-0047] Wilder‐Smith, A. , Vannice, K.S. , Hombach, J. , Farrar, J. and Nolan, T. (2016) Population perspectives and World Health Organization recommendations for CYD‐TDV dengue vaccine. J. Infect. Dis. 214, 1796–1799. 27496977 10.1093/infdis/jiw341

[pbi12869-bib-0048] Williams, K.L. , Wahala, W.M. , Orozco, S. , de Silva, A.M. and Harris, E. (2012) Antibodies targeting dengue virus envelope domain III are not required for serotype‐specific protection or prevention of enhancement in vivo. Virology, 429, 12–20. 22537810 10.1016/j.virol.2012.03.003 PMC3683589

[pbi12869-bib-0049] Yauch, L.E. , Zellweger, R.M. , Kotturi, M.F. , Qutubuddin, A. , Sidney, J. , Peters, B. , Prestwood, T.R. *et al*. (2009) A protective role for dengue virus‐specific CD8^+^ T cells. J. Immunol. 182, 4865–4873. 19342665 10.4049/jimmunol.0801974 PMC2674070

[pbi12869-bib-0050] Zhang, Q. , Bernatoniene, J. , Bagrade, L. , Pollard, A.J. , Mitchell, T.J. , Paton, J.C. and Finn, A. (2006) Serum and mucosal antibody responses to pneumococcal protein antigens in children: relationships with carriage status. Eur. J. Immunol. 36, 46–57. 16342325 10.1002/eji.200535101

[pbi12869-bib-0051] Zhang, Q. , Bagrade, L. , Bernatoniene, J. , Clarke, E. , Paton, J.C. , Mitchell, T.J. , Nunez, D.A. *et al*. (2007) Low CD4 T cell immunity to pneumolysin is associated with nasopharyngeal carriage of pneumococci in children. J. Infect. Dis. 195, 1194–1202. 17357058 10.1086/512617

