# Supplementary material for: Plant‐expressed Fc‐fusion protein tetravalent dengue vaccine with inherent adjuvant properties
Source: Plant Biotechnol J. 2018 Feb 1;16(7):1283–94. doi: 10.1111/pbi.12869 (PMC5999314; doi:10.1111/pbi.12869)
Supplement: Supplementary file 1 — Figure S1 Comparative analysis of D‐PIGS expressed in wild type and ∆XF Benthamiana plants. Figure S2 Temperature stability of high molecular weight D‐PIGS. Figure S3 Comparative analysis of low and high molecular weight D‐PIGS. Figure S4 Dengue virus Neutralization curves obtained with sera from mice immunised with cEDIII antigen alone or in combination with Alum. Figure S5 Gating strategy for analysing T‐cell intracellular cytokine staining by flow cytometry. Figure S6 Gating strategy for analysing tonsillar T‐cell proliferative response by CFSE staining and flow cytometry. Table S1 Kinetics data for D‐PIGS interactions with IgG Fc receptors by surface plasmon resonance. Table S2 Time to 50% dissociation of antibody analyte from receptor ligand. Appendix S1 Functional characterisation of D‐PIGS by C1q ELISA and cell surface binding; protocol description. Appendix S2 Humoral responses in sera of immunized mice; protocol description. Appendix S3 T‐cell proliferation and IFN‐γ; protocol description. [file PBI-16-1283-s001.docx]

**Plant-expressed Fc-fusion protein tetravalent dengue vaccine with inherent adjuvant properties**

Mi Young Kim^1,2^, Alastair Copland^1^, Kaustuv Nayak^3^, Anmol Chandele^3^, Muhammad Shamsher Ahmed^4^, Qibo Zhang^4^, Gil Reynolds Diogo^1^, Matthew John Paul^1^, Sven Hofmann^1^, Moon-Sik Yang^2^, Yong-Suk Jang^2^, Julian KC Ma^1,^* and Rajko Reljic^1,^*

1. Institute for Infection and Immunity, St George’s University of London, SW 17 0RE, UK
2. Department of Molecular Biology and The Institute for Molecular Biology and Genetics, Chonbuk National University, Jeonju 54896, Korea
3. ICGEB-Emory Vaccine Center, International Center for Genetic Engineering and Biotechnology,, Aruna Asaf Ali Marg, Vasant Kunj, New Delhi, India
4. Department of Clinical Infection, Microbiology and Immunology, Institute of Infection and Global Health, University of Liverpool, United Kingdom

*Corresponding authors:

[rreljic@sgul.ac.uk](mailto:rreljic@sgul.ac.uk)

[jma@sgul.ac.uk](mailto:jma@sgul.ac.uk)

Institute for Infection and Immunity, St George’s University of London, UK

London SW17 0RE, UK

Tel: +208-725 0554

Fax: +0208-725 3487

Fig.S1


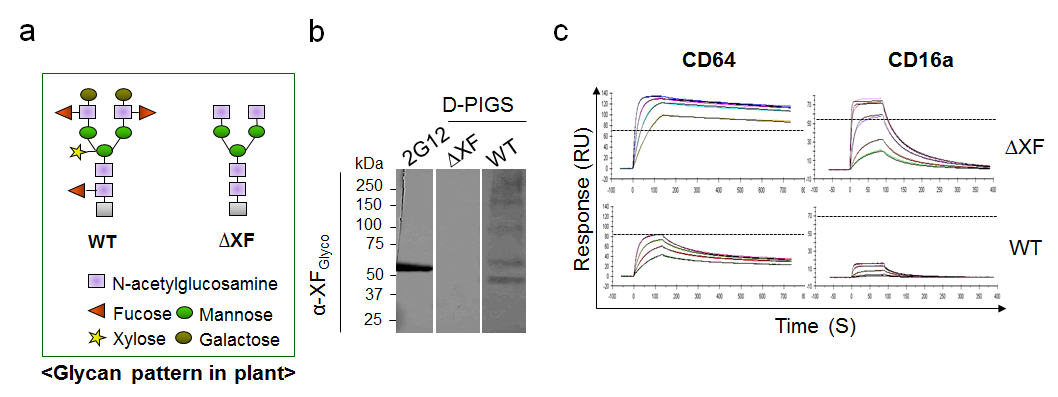


Fig S1: Comparative analysis of D-PIGS expressed in wild type and ∆XF Benthamiana plants

1. Schematic showing difference in glycosylation
2. Detection of D-PIGS glycan moiety in crude plant extracts. Lane, 2G12; monoclonal anti-HIV antibody expressed in wild type and used as positive control; ∆XF; D-PIGS lacking fucose and xylose: WT; D-PIGS expressed in wild type plants
3. Differential binding of wild type and ∆XF plant derived D-PIGS to CD64 and CD16a by SPR

Fig.S2


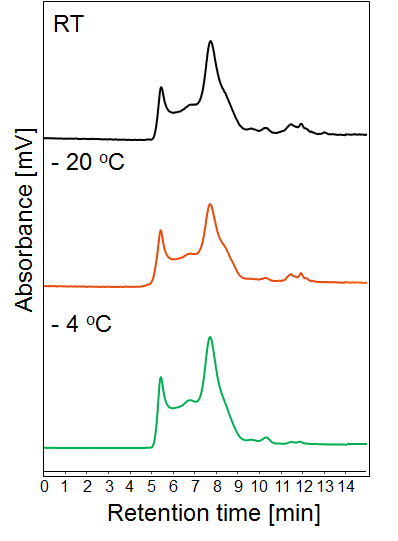


Fig.S2: Temperature stability of high molecular weight D-PIGS. High molecular fraction was stored at indicated temperatures for 24 h and re-analysed by HPLC for possible degradation or changes in molecular profiles.


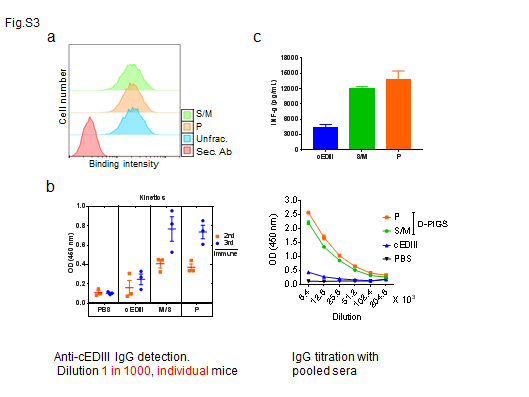


Fig.S3: Comparative analysis of low and high molecular weight D-PIGS.

1. Binding to U937 cells by flow cytometry analysis; S/M, single chain/monomer; P, polymer; ‘Unfract’, unfractionated D-PIGS50 μg of protein was incubated with 1 million cells.
2. Anti-cEDIII IgG response in immunised mice. On the left are shown individual mouse IgG responses following second and third immunisation (for 1:1000 dilution); On the right are shown IgG titration curves for pooled mouse sera from each immunisation group.
3. IFN-γ in 3-day splenic cell culture supernatants following antigen stimulation.

Fig.S4


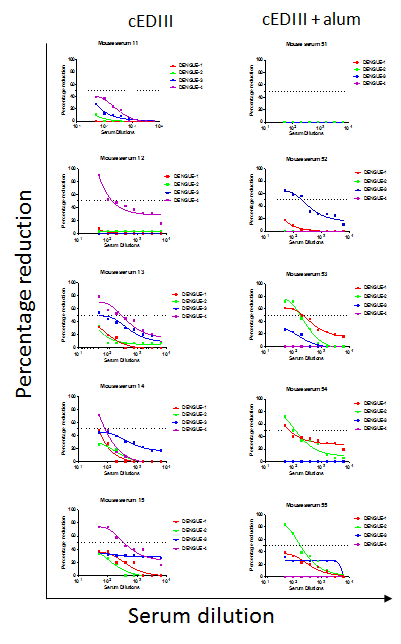


**Fig.S4: Neutralising activity of cEDIII and cEDIII + alum immune sera from mice**

Shown are the FRNT scatter plots and the titration curves from serial dilutions of immune sera from 5 mice for cEDIII alone and for cEDIII + alum. 50 % neutralisation cut off (perforated line) is indicated

Fig.S5


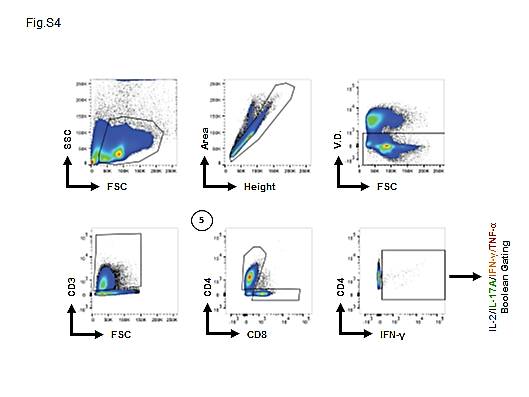


Fig.S5: Gating strategy for analysing T-cell intracellular cytokine staining by flow cytometry.

Fig.S6

**Fig.S6:** Gating strategy for analysing tonsillar T-cell proliferative response by CFSE staining and flow cytometry.

**Supplementary table S1.** Kinetics data for D-PIGS interactions with IgG Fc receptors by surface plasmon resonance

| **Analyte** | **CD16a** | | | | | **CD64** | | | |
| --- | --- | --- | --- | --- | --- | --- | --- | --- | --- |
|  | **k_a_^1^ (/Ms)** | **k_d_^1^**  **(/s)** | **k_a_^2^**  **(/Ms)** | **k_d_^2^**  **(/s)** | **K_D_**  **(M)** | | **K_a_**  **(/Ms)** | **K_d_**  **(/s)** | **K_D_**  **(M)** |
| **hIgG1** | 2.114x10^5^ | 0.1125 | 0.001961 | 0.003627 | 3.453x10^-7^ | | 5.856x10^5^ | 1.073x10^-4^ | 1.832x10^-10^ |
| **D-PIGS** | 7.889 x10^5^ | 0.02909 | 0.004855 | 0.009970 | 2.480x10^-8^ | | 7.483x10^5^ | 2.293x10^-4^ | 3.064x10^-10^ |

**Supplementary table S2.** Time to 50% dissociation of antibody analyte from receptor ligand.

| **Ligand** | **Analyte** | **Time to 50% Dissociation** |
| --- | --- | --- |
| **CD64** | D-PIGS (unfractionated)  D-PIGS (polymers)  D-PIGS (M/S)  hIgG1 | >600s  >600s  >600s  >600s |
| **CD32a** | D-PIGS (unfractionated)  D-PIGS (polymers)  D-PIGS (M/S)  hIgG1 | >600s  >600s  10s  <5s |
| **CD16a** | D-PIGS (unfractionated)  D-PIGS (polymers)  D-PIGS (M/S)  hIgG1 | >600s  >600s  61s  58s |

**Supplementary text S1**

**Functional characterisation of D-PIGS by C1q ELISA and cell surface binding**

A C1q complement binding ELISA was performed to compare the binding of monomeric and polymeric D-PIGS. 10 μg/mL of human C1q (Calbiochem) was coated onto ELISA plates and after 12 h incubation and blocking (5% non-fat dry milk protein solution in PBS), 2-fold serial dilutions of samples were added. Following further incubation (2 h at 37^o^C), peroxidase-conjugated anti-mouse IgG antiserum was used as the detection antibody with Sigma OPD peroxidase substrates. Absorbance was determined at 450 nm using a Sunrise plate reader (Tecan, UK). To test the capacity of D-PIGS to bind to Fc-receptor bearing cells, U937 monocytes (ATCC) grown in RPMI medium supplemented with 10% Foetal Bovine Serum (FBS) were used; 1 million cells were suspended in 100 μL 3 % BSA in PBS buffer and incubated on ice for 2 h with 10 μg/mL of D-PIGS. Unbound protein was removed by washing 3 times in binding buffer and secondary antibody [anti-human IgG-FITC antiserum (The Binding Site)] added. Cells were analysed for green fluorescence in a Becton-Dickinson flow cytometer.

**Supplementary text S2**

**Humoral response**

cEDIII-specific IgG antibody responses induced by D-PIGS were tested by ELISA, as described previously ([Kim et al., 2017](#_ENREF_22)). Briefly, ELISA plates were coated with cEDIII antigen (5 μg/ml) and probed with either 1000-fold diluted mouse sera (for monitoring response after each immunisation) or by 5-fold serial dilutions (end point of the experiment). Antigen-specific IgG, IgG1 and IgG2a responses were detected by peroxidase-conjugated sheep secondary antibodies (The Binding Site).

**Supplementary Text S3**

**T-cell proliferation and IFN-γ**

To measure T-cell proliferation, splenocytes were stimulated with 5 μg/mL cEDIII for 6 days. Soluble anti-CD3 antibody (1 μg/mL; Biolegend) was used as a positive control. Cells were then stained with viability dye alongside Fc receptor blockade identically to the polyfunctionality assays. A surface stain of CD8-Brilliant Violet 510, CD44-FITC and CD62L-PE (all from Biolegend) was performed for 45 minutes at 4°C. The stained cells were then fixed using the eBioscience FOXP3 Staining Kit according to the manufacturer’s instructions, and an intracellular stain of Ki67-APC (45 minutes at 4°C) was performed in order to detect proliferating cells. FMOs were used to determine gating boundaries. Cells were washed and then acquired on a BD FACSCanto II. IFN-γ was quantified in stimulated culture supernatants by ELISA, using mouse Th1/Th2 ELISA Ready-SET-Go kit; affymetrix eBioscience, USA), according to manufacturer’s instructions. For tonsillar cell cultures, cell proliferation was examined by CFSE (5(6)-Carboxyfluorescein N-hydroxysuccinimidyl ester) staining of mononuclear cells (Molecular Probes, UK).
